# PBT2 potentiates carbapenem antibiotics against diverse *Klebsiella pneumoniae* strains

**DOI:** 10.1128/spectrum.02109-26

**Published:** 2026-09-25

**Authors:** Aimee Tan, Abigail R. Hartono, Adam P. Gunn, Claire L. Gorrie, David M. P. De Oliveira, Francesca L. Short, Mark J. Walker, Norelle L. Sherry, Benjamin P. Howden, Christopher A. McDevitt

**Affiliations:** 1 Department of Microbiology and Immunology at the Peter Doherty Institute for Infection and Immunity, University of Melbourne, Melbourne, Victoria, Australia; 2 Centre for Pathogen Genomics, University of Melbourne, Melbourne, Victoria, Australia; 3 Microbiological Diagnostic Unit (MDU) Public Health Laboratory, Department of Microbiology and Immunology at the Peter Doherty Institute for Infection and Immunity, University of Melbourne, Melbourne, Victoria, Australia; 4 Centre for Superbug Solutions, Institute for Molecular Bioscience, The University of Queensland 1974, Brisbane, Queensland, Australia; 5 Department of Microbiology, Infection Program, Biomedicine Discovery Institute, Monash University, Clayton, Victoria, Australia; 6 Department of Infectious Diseases and Immunology, Austin Health 740438, Heidelberg, Victoria, Australia; 7 Department of Microbiology, Royal Melbourne Hospital, Parkville, Victoria, Australia; Universita degli Studi Roma Tre, Roma, Italy

**Keywords:** antibiotic resistance, antimicrobial, ESKAPE, ionophore, therapeutic, *Klebsiella*, carbapenem

## Abstract

Carbapenem-resistant *Klebsiella pneumoniae* is recognized by the World Health Organization as a critical priority for urgent antimicrobial development. Due to the lack of novel antimicrobial classes in the development pipeline, this study investigated the application of an antibiotic potentiator, the zinc ionophore PBT2, to restore the utility of critical antibiotics. The data show that zinc and PBT2 can increase the sensitivity of diverse clinical strains of *K. pneumoniae* to carbapenem antibiotics, including those strains containing metallo-β-lactamases and porin mutations. PBT2 induced zinc and iron dyshomeostasis in these strains, resulting in altered uptake of multiple antimicrobial agents. Furthermore, PBT2 exposure also decreased resistance to agents, including ethanol and hydrogen peroxide, and reduced the potential for horizontal gene mobilization. Collectively, these impacts on metal homeostasis and cell composition suggest that PBT2 may have potential as an adjunct therapy for the treatment of multidrug-resistant *K. pneumoniae*.

**IMPORTANCE:**

Carbapenem-resistant *Klebsiella pneumoniae* is a major nosocomial threat and a top World Health Organization priority for urgent antimicrobial development. Resistance can be mediated by multiple classes of mobilizable carbapenemase enzymes, with varied treatment recommendations, depending on which class is present. This work describes how a repurposed drug called PBT2 can potentiate carbapenem activity against a clinical *K. pneumoniae* panel, independent of resistance mechanism. This could support the development of PBT2 as a broad-spectrum potentiator compound against drug-resistant *K. pneumoniae*.

## INTRODUCTION

*Klebsiella pneumoniae* is a leading cause of healthcare-acquired infections, including hospital- and ventilator-acquired bacterial pneumonia and bloodstream, device-, and surgical-site infections (1). In clinical settings, these serious opportunistic *K. pneumoniae* infections are frequently preceded by asymptomatic colonization of the gastrointestinal tract (2, 3), with host dysbiosis or antibiotic treatment enabling the outgrowth of strains with antimicrobial resistance (AMR) determinants that can disseminate and cause disease (4, 5). Consequently, the AMR profile of this pathogen is of critical concern due to the threat posed to healthcare systems, with carbapenem-resistant *K. pneumoniae* (CrKp) recently declared by the World Health Organization as the top priority for antimicrobial development (6). Carbapenems are currently intravenous, hospital-only antibiotics on the “watch” list of antimicrobials, prioritized for stewardship programs and resistance monitoring (7). Despite these measures, carbapenem resistance is already widespread in some areas, with rates reaching as high as 60% of invasive isolates in countries including Italy and Greece (1). CrKps are the leading cause of carbapenem-resistant infections worldwide and are conservatively estimated to have caused 1.59 million serious infections and 270,000 bloodstream infections in 2019 (8), underscoring the urgent need for new interventions.

Resistance to carbapenems in Gram-negative pathogens is primarily mediated by enzymatic inactivation. Carbapenemase determinants are structurally diverse and classified into the Ambler β-lactamase classes A (e.g., *bla*_KPC_), B (*bla*_NDM_, *bla*_IMP_, and *bla*_VIM_), and D (certain *bla*_OXA_ alleles), with the class B metallo-β-lactamase enzymes dependent on zinc for activity (9). Many carbapenemase genes are encoded on mobile genetic elements, enabling dissemination in response to selective pressure in healthcare settings. *K. pneumoniae* is a key contributor to the evolution and dissemination of carbapenemases both within the species and to other pathogens, including *Escherichia coli*, *Acinetobacter baumannii*, and *Pseudomonas aeruginosa* (1, 10). AMR can be further augmented by mutational variations that either reduce the permeability of the cell to the drug or increase the expression of efflux pumps (11). In *K. pneumoniae*, carbapenem resistance is frequently associated with alterations in the OmpK35 and OmpK36 porins (12, 13), while efflux pumps such as AcrAB-TolC (14) appear to play a more limited role, despite previous associations with *K. pneumoniae* antibiotic resistance (15, 16).

To combat carbapenem resistance, co-therapeutic strategies are increasingly employed, typically involving co-administration of antibiotics with β-lactam inhibitors. However, most available inhibitors are class specific and show limited efficacy against the class B metallo-β-lactamase enzymes (17). Thus, while new combinations show promise, there is still an urgent need to identify further therapeutic strategies, ideally with broad activity profiles against the diverse mechanisms and genetic backgrounds that drive resistance in *K. pneumoniae*.

This study investigated the potential of PBT2, a neurodegenerative therapeutic repurposed as a potentiator of carbapenem antibiotics against *K. pneumoniae*. PBT2 was originally developed as a treatment for Alzheimer’s disease (18), with recent discoveries highlighting that its ionophore properties could dysregulate bacterial zinc homeostasis and compromise resistance to antibiotics. When co-administered with antibiotics, PBT2 has shown synergistic activity across multiple drug classes, including polymyxins (in *K. pneumoniae*, *E. coli*, and *Acinetobacter baumannii* [19]; *Streptococcus pyogenes* and *Staphylococcus aureus* [20]), tetracyclines (in *A. baumannii* [21] and *Neisseria gonorrhoeae* [22]), tigecycline (in *A. baumannii* [21] and *K. pneumoniae* [23]), and ampicillin (*S. pneumoniae* [24]). Co-therapeutic administration of PBT2 with polymyxins also significantly reduced the burden of bacterial disease in murine models of pneumonia (24) and sepsis (19), indicating potential utility against severe respiratory and bloodstream infections caused by CrKp.

Here, we show that PBT2 reduces carbapenem resistance by up to 16-fold against a panel of *K. pneumoniae* isolates, encompassing representatives from all Ambler carbapenemase classes. This effect is mediated through PBT2-induced dysregulation of metal homeostasis, mirroring the zinc intoxication response of *K. pneumoniae*. This dysregulation leads to membrane perturbation, likely increasing antimicrobial agent permeability, and perturbing the conjugative transfer of DNA.

## RESULTS AND DISCUSSION

### PBT2 and zinc increase carbapenem sensitivity in diverse AMR *K. pneumoniae* clinical isolates

This study identified a panel of five contemporary clinical *K. pneumoniae* isolates representing a cross-section of *K. pneumoniae* with diverse drug resistance mechanisms, including carbapenemase representatives from Ambler classes A (*bla*_KPC-2_), B (*bla*_IMP_*, bla*_NDM_), and D (*bla*_OXA-48_ and *bla*_OXA-181_), porin mutations, and other resistance determinants (Table S1). This *K. pneumoniae* strain panel, along with the well-characterized reference strains AJ218 and B5055, from classical and hypervirulent lineages, respectively, were assessed for the minimum inhibitory concentration (MIC) of PBT2 to inhibit growth in the absence and presence of subinhibitory zinc concentrations (0–512 µM ZnSO_4_, to reflect a range of concentrations found in the host) in cation-adjusted Mueller–Hinton broth. Data from broth microdilution checkerboard assays show that PBT2 perturbed growth of all isolates, with the determined MICs inversely proportional to the concentration of supplemented zinc. Specific MICs varied between strains, with the lowest susceptibility observed for the MS6671 strain, consistent with previous studies (19), suggesting that *K. pneumoniae* genetic background influences PBT2 susceptibility (Table S2).

Building on the above framework, we next assessed the impact of PBT2 on carbapenem resistance within the strain panel in low and supplemented zinc backgrounds. The carbapenem concentration needed to inhibit growth in the presence of 256 µM ZnSO_4_ and/or a subinhibitory concentration of PBT2 (0.5× MIC, as determined above) was quantified by broth microdilution MIC assays. This revealed that carbapenem resistance levels differed between strains, consistent with the distinct genetic bases of resistance. In low zinc conditions, PBT2 did not decrease the carbapenem MIC for the respective *K. pneumoniae* isolates. In zinc-supplemented conditions, isolates encoding zinc-dependent metallo-β-lactamases (Kp661, Kp705, and Kp706) had increased carbapenem resistance, attributable to the increased zinc availability in the medium. The combination of PBT2 and zinc increased *K. pneumoniae* susceptibility to carbapenems by 2- to 16-fold, relative to these control conditions (Table 1). Together, these findings highlight the capacity of PBT2 to potentiate carbapenem activity across a diverse panel of CrKp clinical isolates.

**TABLE 1:** MICs for carbapenems against clinical *K. pneumoniae* strains in the presence of PBT2 and ZnSO_4_

Strain	Carbapenem resistance determinants^*a*^	Assay conditions^*b*^					
		PBT2 (µM)	Antibiotic	Low zinc (5.05 µM ZnSO_4_)		Zinc supplemented (256 µM ZnSO_4_)	
				−PBT2	+PBT2	−PBT2	+PBT2
AJ218	None applicable	8	Imipenem	**0.5**	**0.5**	**0.5**	**0.125**
			Meropenem	**0.0625**	**0.0625**	**0.0625**	**0.03125**
B5055	None applicable	4	Imipenem	**2**	**2**	**2**	**0.5**
			Meropenem	**0.125**	**0.125**	**0.125**	**0.0625**
MS6671	OXA-181 (three copies), OmpK35 (76%), OmpK36TD	64	Imipenem	8	8	8	**2**
			Meropenem	32	32	32	8
Kp125	KPC-2, OmpK35 (25%)	32	Imipenem	64–128	64	128	32
			Meropenem	64–128	64	64–128	**4**–8
Kp661	NDM-5, OXA-48, OmpK36GD	16	Imipenem	128	128	512	64
			Meropenem	512	512	512	64
Kp705	NDM-1, OXA-181, OmpK35 (40%)	32	Imipenem	2–4	4	4	**1**
			Meropenem	**4**	**4**	**4**	**1**
Kp706	IMP-4 (homolog), OmpK35 (79%)	8	Imipenem	8	8	16–32	**2**
			Meropenem	16	16	16	**<0.5**

^*a*^ Carbapenem resistance determinants from genome sequence data (accession numbers listed in Table S1) and confirmed using Pathogenwatch (25).

^*b*^ MIC values (µg·mL^−1^) as determined by broth microdilution assays in normal cation-adjusted Mueller–Hinton broth (no ZnSO_4_, no PBT2 column) and with supplementation as indicated (ZnSO_4_ at 256 µM and PBT2 at concentrations listed), with the modal value presented (from *n* ≥ 3). Cells are shaded in gray where PBT2 decreases MICs ≥4-fold compared to the relative −PBT2 control, and MICs below resistance breakpoints are shown in bold (based on European Committee on Antimicrobial Susceptibility Testing resistance breakpoints for imipenem [≥4 µg·mL^−1^] and meropenem [≥8 µg·mL^−1^]) (26).

### PBT2 is effective against horizontally transferred resistance

**Fig 1:**
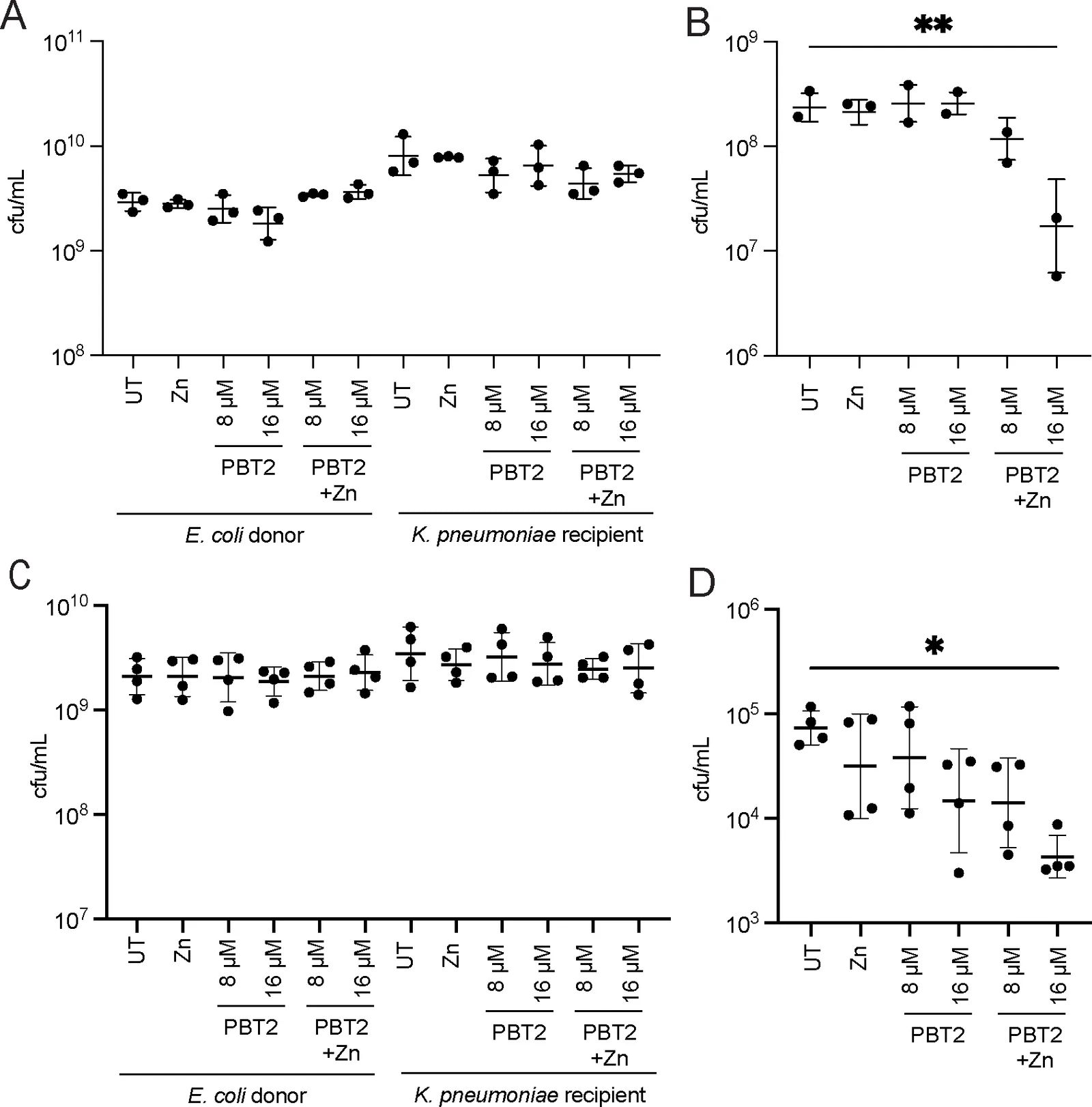
Impact of PBT2 and zinc on horizontal gene transfer in *K. pneumoniae*. (**A**) Survival of *E. coli* JKE201 pBHR1 and *K. pneumoniae* AJ218 after 1 h incubation on Luria agar supplemented with Zn and PBT2 at the concentrations indicated. (**B**) Number of transconjugants isolated from 1:1 JKE201 pBHR1:AJ218 conjugations, post-incubation on Luria agar supplemented with Zn and PBT2 at the concentrations indicated. (**C**) Survival of *E. coli* JKE201 pDS1028 and *K. pneumoniae* after 1 h incubation on Luria agar supplemented with Zn and PBT2 at the concentrations indicated. (**D**) Number of transconjugants isolated from 2:1 JKE210 pDS1028:AJ218 conjugations, post-incubation on Luria agar supplemented with Zn and PBT2 at the concentrations indicated. Statistical significance of differences was determined by comparison to Luria alone, by one-way ANOVA analysis with Dunnett post-test. ns, not significant; *, *P* < 0.05; **, *P* < 0.01.

We then examined whether PBT2 maintained activity against mobilizable carbapenem resistance determinants and in different *K. pneumoniae* genetic backgrounds. Initial attempts to assess the impact of PBT2 on horizontal gene transfer of native resistance plasmids from clinical *K. pneumoniae* into the drug-sensitive strains were unsuccessful under all conditions tested (regardless of the presence or absence of PBT2). Accordingly, transfer of the pBHR1 conjugative plasmid from an *E. coli* donor, JKE201, was assessed instead. These assays showed that while donor and recipient strains maintain similar viability under all tested conditions (Fig. 1A), a significant 1-log reduction in conjugants was produced by assays performed on media containing 16 µM PBT2 with zinc supplementation, relative to control conditions (Fig. 1B). Similar results were observed for assays conducted with a conjugative transposon vector (with resistance carried on an integrative transposon conjugated via plasmid pDS1028, from *E. coli* JKE210 into *K. pneumoniae* AJ218; Fig. 1C and D). Together, these data suggest that PBT2-mediated zinc stress may inhibit horizontal DNA transfer efficiency and the movement of AMR determinants, consistent with other reports on the impact of zinc ionophores in other enteric bacteria (27).

To investigate the impact of PBT2 on carbapenemases transferred into independent genetic backgrounds, carbapenemase alleles were cloned into the pACYC184 backbone for constitutive expression using the tetR promoter and transformed into the representative *K. pneumoniae* strains AJ218 (classical) and B5055 (hypervirulent). In standardized conditions, strains carrying the *bla*_IMP_ and *bla*_OXA_ alleles were unable to mediate resistance to meropenem (Table S3), consistent with prior observations that some carbapenemases require additional core genome mutations to mediate clinical resistance (28). Introduction of *bla_KPC-2_* modestly elevated carbapenem resistance, although still below the European Committee on Antimicrobial Susceptibility Testing resistance breakpoint (>8 µg·mL^−1^ meropenem) (26). Nevertheless, this resistance was reduced by treatment with zinc and PBT2 (Table S3). Strains with introduced *bla_NDM-1_* and *bla_NDM-5_* zinc-dependent carbapenemases showed substantial activity against carbapenems, with the highest resistance seen in zinc-supplemented media. Addition of PBT2 to unsupplemented media did not impact MIC values, but the combination of zinc and PBT2 reduced carbapenem resistance two- to eightfold by comparison to zinc supplementation alone (Table 2). Taken together, these data suggest that PBT2 can still alter carbapenem susceptibility when resistance determinants have been horizontally transferred into susceptible genetic backgrounds.

**TABLE 2:** MICs for carbapenems against strains with introduced carbapenemase resistance genes in the presence of PBT2 and ZnSO_4_

Plasmid	Strain	Assay conditions^*a*^					
		PBT2	Antibiotic	Low zinc (5.05 µM ZnSO_4_)		Zinc supplemented (256 µM ZnSO_4_)	
				−PBT2	+PBT2	−PBT2	+PBT2
p*bla_NDM-1_*	B5055	4	Imipenem	4	4	16	**2**
			Meropenem	**4**	**2**	64	16
	AJ218	8	Imipenem	4	4	16	8
			Meropenem	**4**	**4**	32	8–16
	∆OmpK35	8	Imipenem	4	4	16	8
			Meropenem	**4**	**4**	32	8
	∆OmpK36	8	Imipenem	8	8	64	8
			Meropenem	4−8	**4**	32−64	32
	∆OmpK35 ∆OmpK36	8	Imipenem	64	64	512	128
			Meropenem	32	64−128	256	128
p*bla_NDM-5_*	B5055	4	Imipenem	8	4	32	**2**
			Meropenem	16−32	**4**	64−128	16
	AJ218	8	Imipenem	4	4	32	4
			Meropenem	16	16	32−128	16
	∆OmpK35	8	Imipenem	4	8	32	4
			Meropenem	32	16	64	64
	∆OmpK36	8	Imipenem	8	8	64	4
			Meropenem	16	16	128	128
	∆OmpK35 ∆OmpK36	8	Imipenem	128	128	512	128
			Meropenem	256	256	256	256

^*a*^ MIC values (µg·mL^−1^) as determined by broth microdilution assays in normal cation-adjusted Mueller–Hinton broth (no ZnSO_4_, no PBT2 column) and with treatments as indicated (ZnSO_4_ at 256 µM and PBT2 at the concentrations listed), with the modal value presented (from *n* ≥ 3). Cells are shaded in gray where PBT2 decreases MICs ≥4-fold compared to the relative −PBT2 control, and MICs below resistance breakpoints are shown in bold (based on European Committee on Antimicrobial Susceptibility Testing resistance breakpoints for imipenem [≥4 µg·mL^−1^] and meropenem [≥8 µg·mL^−1^]) (26).

### PBT2 carbapenem potentiation is independent of specific resistance mechanisms

**Fig 2:**
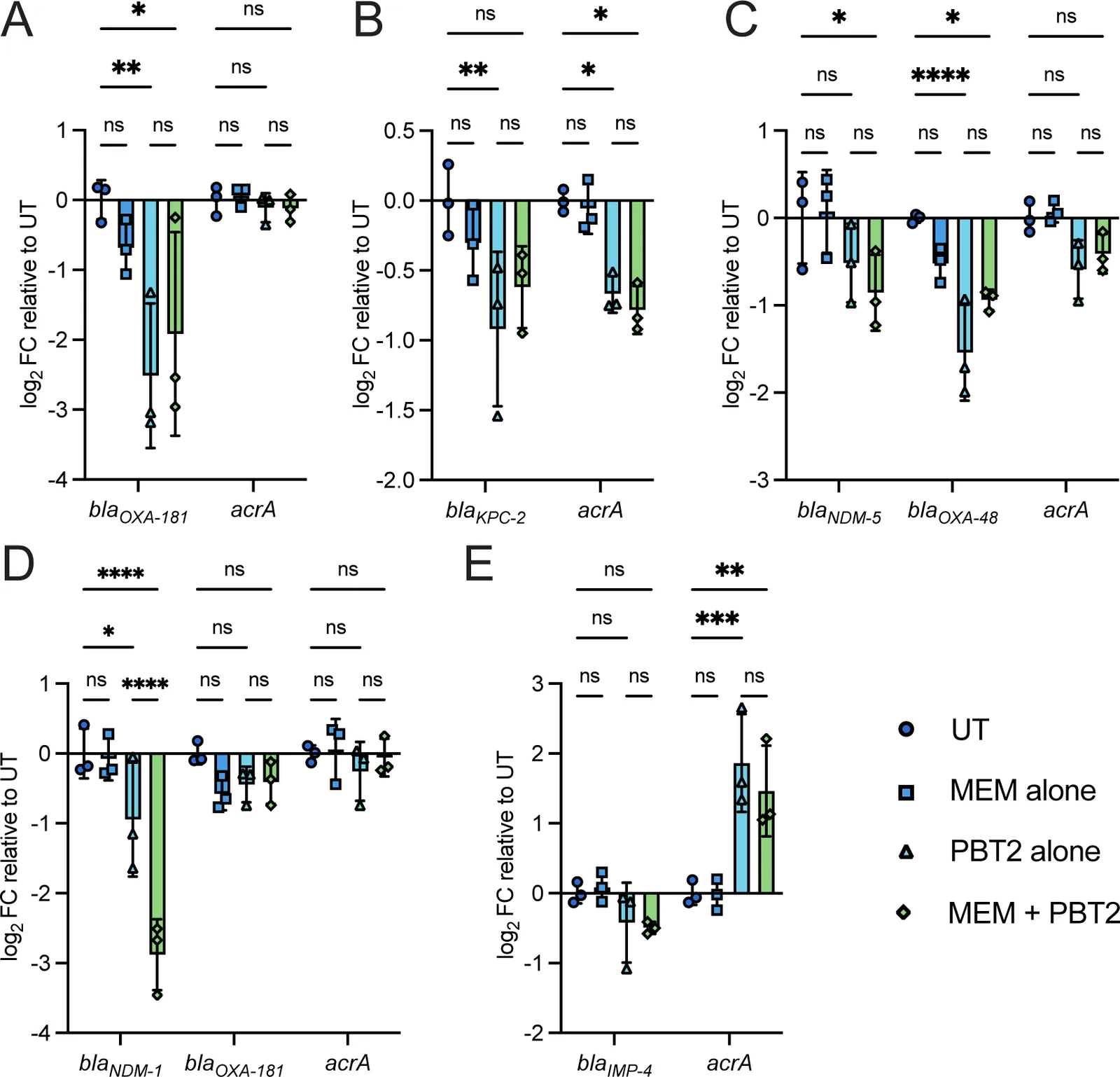
Transcriptional quantification of *K. pneumoniae* carbapenem resistance mechanisms in response to subinhibitory PBT2 and meropenem supplementation. Quantitative real-time PCR analysis of carbapenem resistance determinants (carbapenemases and efflux pump *acrA*) for MS6671 (**A**), Kp125 (**B**), Kp661 (**C**), Kp705 (**D**), and Kp706 (**E**) strains grown in zinc-supplemented CAMHB with or without subinhibitory PBT2 (0.5× MIC, 256 µM ZnSO_4_) and meropenem (0.25× MIC, relative to PBT2 and ZnSO_4_ conditions). Data are shown as mean (±SD) log_2_ fold change in gene expression in PBT2 and meropenem treatment conditions compared to the untreated (UT) condition, normalized to *rpoD* (*n* = 3). Statistical significance of differences was determined by two-way ANOVA analysis with Tukey post-test. ns, not significant; *, *P* < 0.05; **, *P* < 0.01; ***, *P* < 0.001; ****, *P* < 0.0001.

To explore how PBT2 and zinc modulate carbapenem sensitivity, the impact of PBT2 on resistance gene expression was assessed. Here, *K. pneumoniae* was grown in zinc-supplemented media, with or without subinhibitory concentrations of PBT2 and meropenem. The data showed that carbapenemase gene expression varied between strains with baseline expression not significantly altered by exposure to meropenem (Fig. 2). Treatment with PBT2 did not definitively affect carbapenemase expression. Although some conditions did show significant reduction of expression in the presence of PBT2 relative to the untreated (no PBT2, no meropenem) condition, these trends were not consistent between strains or carbapenemase types (Fig. 2). Similarly, investigation of *acrB*, a component of the AcrAB-TolC efflux pump that has been previously shown to be impacted by PBT2 (23), showed variable expression across strains, with increased expression in Kp706 but decreased expression in Kp125 in the presence of PBT2 (Fig. 2). Together, these data suggest that while PBT2-mediated suppression of AMR determinant expression may contribute to potentiation of carbapenem activity, it is unlikely to be the sole contributing factor.

Carbapenem resistance is also strongly influenced by cell permeability, with porin mutations facilitating higher resistance levels by reducing the rate of drug penetration below the rate of β-lactam hydrolysis by the cell (13, 28). Given that these resistance phenotypes are caused by mutational inactivation, we did not assess expression levels of the porin mutations in the clinical panel. Instead, we assessed how PBT2- and zinc-mediated carbapenem potentiation was altered in *K. pneumoniae* AJ218 and the isogenic OmpK35 and OmpK36 porin mutant strains. Assessment of the *bla_NDM-1_* and *bla_NDM-5_* carbapenemases transformed into these backgrounds showed that porin mutations increased resistance to carbapenems, with the greatest effect observed in zinc-supplemented media (Table 2). For most conditions tested, the addition of subinhibitory PBT2 to zinc-supplemented media decreased MIC values two- to fourfold relative to the zinc-supplemented condition (Table 2). These data suggest that PBT2 does not require these porins to enter the cell and potentiate antimicrobial activity. The exception to this was in porin mutants expressing NDM-5, wherein meropenem resistance was maintained in the presence of zinc, irrespective of PBT2 treatment (Table 2). This observation contrasts with the impact of PBT2 and zinc on imipenem resistance for these same strains, suggesting PBT2 is still active under these conditions. Accordingly, we infer that PBT2 + zinc is unable to overcome the resistance arising from reduced meropenem penetration and rate of NDM-5 hydrolysis. These data highlight the interplay between antibiotics, AMR determinants, and cell permeability. Nevertheless, the data show that PBT2 + zinc treatment can overcome the reduced antibiotic permeability and increased resistance from porin mutations. Collectively, this suggests that PBT2 increases carbapenem sensitivity via impacts on conserved cellular functions, rather than direct action on resistance mechanisms.

### PBT2 and zinc alter cell permeability and susceptibility to other agents

**Fig 3:**
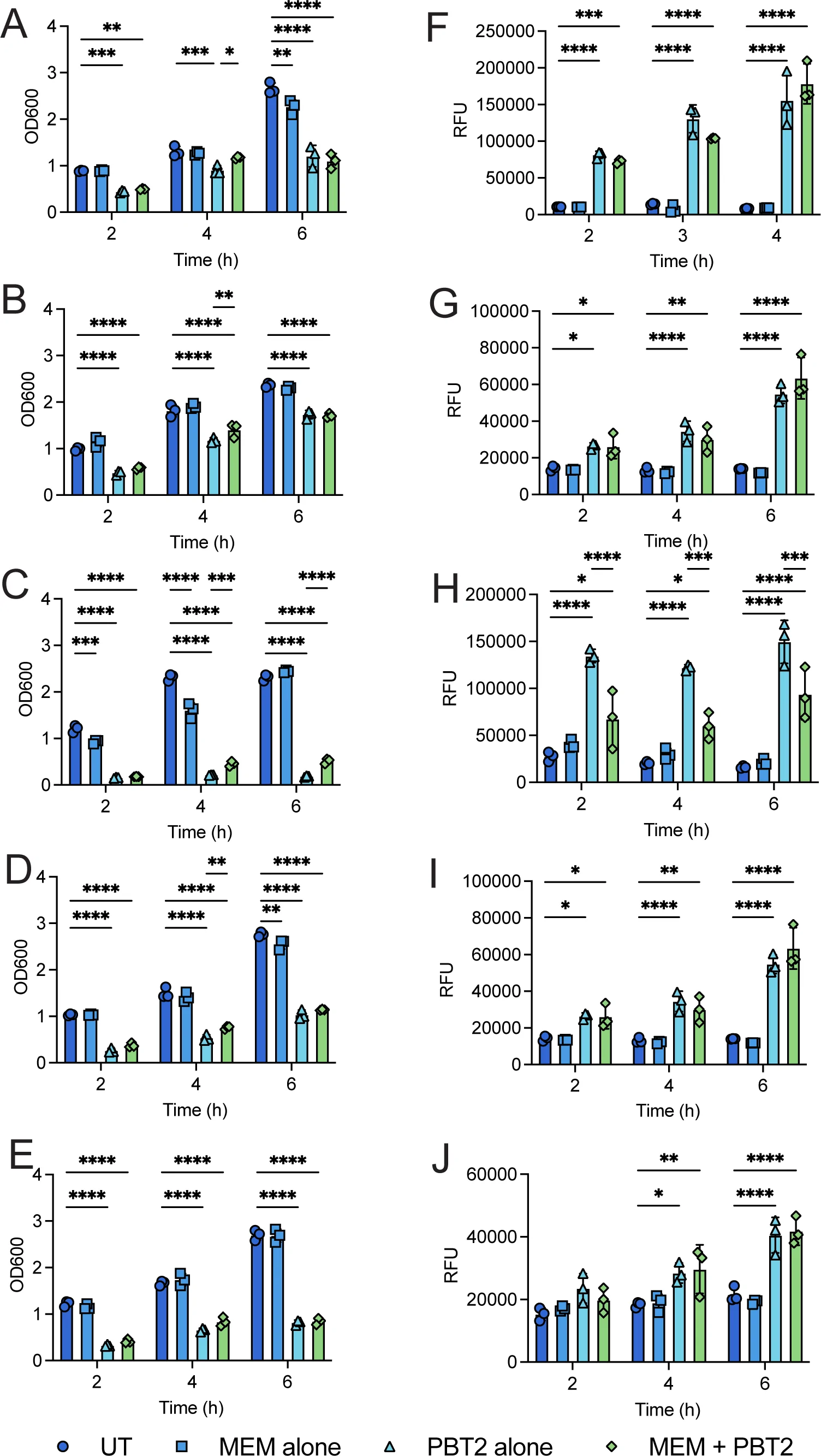
Growth and membrane penetration of cells grown in PBT2 and meropenem. *K. pneumoniae* strains grown in untreated zinc-supplemented media (UT) with or without subinhibitory PBT2 (0.5× MIC, 256 µM ZnSO_4_) and/or meropenem (0.25× MIC, relative to PBT2 and ZnSO_4_ conditions), analyzed for growth (as OD_600_, **A–E**) and relative fluorescence (with relative fluorescence units [RFU] calculated as fluorescence / OD_600_, **F–J**) over time. Data represent values (±SD) from three independent experiments for growth and relative fluorescence for MS6671 (**A and F**), Kp125 (**B and G**), Kp661 (**C and H**), Kp705 (**D and I**), and Kp706 (**E and J**), respectively. Data were obtained from three independent experiments; statistical significance of differences was determined by two-way ANOVA analysis with Tukey post-test. ns, not significant; *, *P* < 0.05; **, *P* < 0.01; ***, *P* < 0.001; ****, *P* < 0.0001.

To further probe the relationship between PBT2 and zinc and the permeability of antimicrobial agents into the cell, differences in membrane penetration and cell death of actively growing *K. pneumoniae* were assessed in zinc-supplemented media, with or without subinhibitory meropenem and PBT2. Assays were performed by inoculating zinc-supplemented media with *K. pneumoniae* strains in the presence and/or absence of subinhibitory PBT2 and meropenem, and cultures were assessed at 2, 4, and 6 h post-inoculation using Sytox Green, a cell impermeant nucleic acid binding dye that emits fluorescence upon DNA binding. The data show that while cultures grown with PBT2 generated less biomass over the duration of the assay (Fig. 3A through E), these cultures had higher relative fluorescence levels (standardized to biomass) relative to untreated conditions (Fig. 3F through J). As subinhibitory meropenem and PBT2 concentrations do not prevent cellular replication (Fig. 3A through E; Fig. S1), this suggests cells remain viable and that fluorescence increases may be attributable to disruptions to cell membranes, which permit dye penetration into the cytoplasm rather than via cell lysis and DNA release.

The potential link between PBT2 and enhanced penetration and activity of antimicrobial agents was further explored via the use of other unrelated antimicrobial agents, namely, ethanol, hydrogen peroxide, and the aminoglycoside antibiotic gentamicin. MIC data showed that strain growth was inhibited by lower concentrations of H_2_O_2_ and ethanol in the presence of subinhibitory PBT2 and zinc, in contrast to either agent alone (Table 3). For gentamicin, substantial variation in resistance was seen across the clinical panel, with higher resistances seen in MS6671, Kp661, and Kp705 compared to Kp125 and Kp706. PBT2 and zinc induced substantial reductions in resistance for three of the strains (Kp125, Kp661, and Kp705) but had no apparent impact on the resistance of the MS6671 and Kp705 strains (Table 3). Notably, these latter two strains express Rmt family ribosome methylases (Table S1), which mediate aminoglycoside resistance via protection of the ribosome target. This raises the possibility that PBT2 was less effective in these strains because this target protection resistance mechanism was less susceptible to changes in drug concentrations than those mediated by drug export or turnover. Collectively, these data indicate that PBT2 + zinc acts non-specifically by altering cell membrane permeability and increasing the penetration of antimicrobial agents. This likely overwhelms the detoxification rate of innate xenobiotic stress resistance mechanisms, whose activity may also be further influenced by PBT2 + zinc exposure.

**TABLE 3:** MICs of agents against clinical strains in the presence of PBT2 and ZnSO_4_

Agent	Strain	Assay conditions^*a*^			
		Low zinc (5.05 µM ZnSO_4_)		Zinc supplemented (256 µM ZnSO_4_)	
		−PBT2	+PBT2	−PBT2	+PBT2
H_2_O_2_ (mM)	MS6671	2.5	1.25	2.5	1.25
	Kp125	2.5	2.5	2.5	1.25
	Kp661	2.5	2.5	2.5	1.25
	Kp705	2.5	2.5	2.5	1.25
	Kp706	2.5	2.5	2.5	1.25
EtOH (%)	MS6671	10	5	5	<2.5
	Kp125	10	5	5	5
	Kp661	10	10	10	2.5
	Kp705	10	10	10	5
	Kp706	10	10	10	5
Gentamicin (µg·mL^−1^)	MS6671	>512	>512	>512	>512
	Kp125	4	2	4	1
	Kp661	256	256	512	8
	Kp705	>512	>512	>512	>512
	Kp706	128	128	128	8

^*a*^ MIC values as determined by broth microdilution assays in normal cation-adjusted Mueller–Hinton broth (no ZnSO_4_, no PBT2 column) and with treatments as indicated (ZnSO_4_ at 256 µM and PBT2 at the concentrations listed in Table 1), with the modal value presented (from *n* ≥ 3).

### PBT2 consistently causes metal dyshomeostasis effects that are linked with carbapenem potentiation

**Fig 4:**
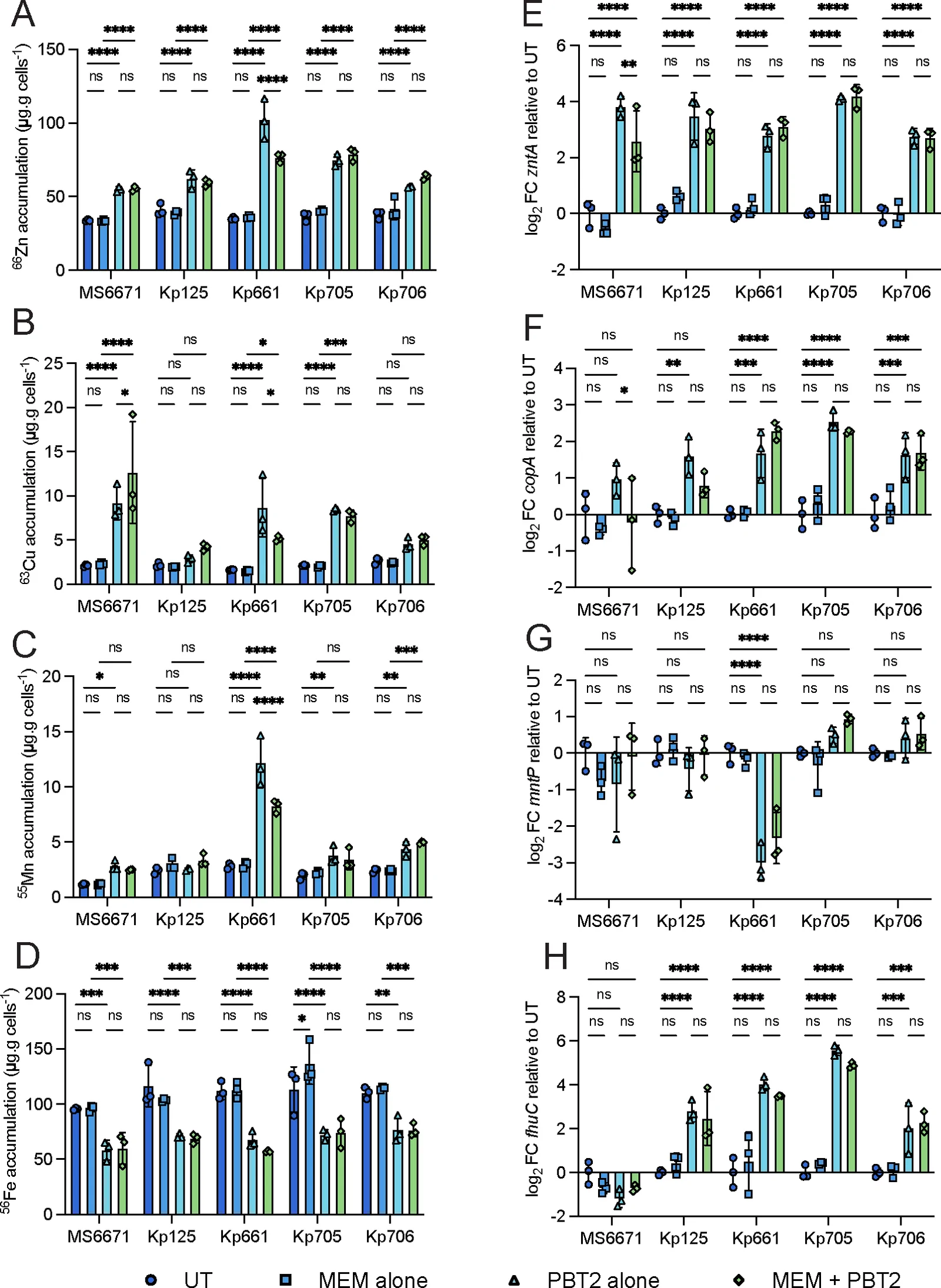
*K. pneumoniae* elemental content analyses and transcriptional quantification in response to PBT2 and meropenem supplementation. *K. pneumoniae* strains grown in untreated zinc-supplemented media (UT) with or without subinhibitory PBT2 (0.5× MIC, 256 µM ZnSO_4_) and/or meropenem (0.25× MIC, relative to PBT2 and ZnSO_4_ conditions), analyzed for whole cell metal content, represented as mean accumulation (±SD) μg metal·g cells^−1^ (dry weight) (**A–D**) and corresponding Quantitative real-time PCR analysis of metal-responsive gene expression shown as mean (±SD) log_2_ fold change in gene expression in PBT2 and meropenem treatment conditions compared to the UT condition, normalized to *rpoD* (**E–H**). Data show the corresponding cellular content and gene expression of ^66^Zn (**A**) and *zntA* (**E**), ^63^Cu (**B**) and *copA* (**F**), ^55^Mn (**C**) and *mntP* (**G**), and ^56^Fe (**D**) and *fhuC* (**H**), respectively. Data were obtained from three independent experiments; statistical significance of differences was determined by two-way ANOVA analysis with Tukey post-test. ns, not significant; *, *P* < 0.05; **, *P* < 0.01; ***, *P* < 0.001; ****, *P* < 0.0001.

PBT2 is an ionophore that has previously been observed to increase cellular zinc and copper, and decrease cellular iron in *K. pneumoniae* MS6671 (19). Here, we investigated how broadly conserved these metal dyshomeostasis impacts were across the *K. pneumoniae* isolate panel. This was addressed using subinhibitory concentrations of PBT2 and/or meropenem in zinc-supplemented media. Growth analyses revealed that the addition of PBT2 delayed growth of cells and limited final biomass in all strains. By contrast, exposure to meropenem only exerted a mild impact, which was not consistently observed across all strains (Fig. S1). Similar impacts were noted at the molecular level, with PBT2, but not meropenem, inducing broadly similar metal content (Fig. 4A through D) and transcriptomic changes (Fig. 4E through H).

The most notable impact of PBT2 on cellular metal content was on zinc, with PBT2 significantly elevating levels for all strains (Fig. 4A). This change correlated with increased transcription of *zntA*, encoding the major *K. pneumoniae* zinc efflux transporter (29), with PBT2 treatment inducing 3–4 log_2_ fold increases in expression relative to untreated strains (Fig. 4E). Increased zinc uptake and upregulation of efflux mechanisms is the most frequently identified impact of PBT2 on bacterial cells (19, 21, 24, 30, 31), with similar impacts present in all *K. pneumoniae* strains. Similarly, PBT2 was associated with a significant increase in copper accumulation (Fig. 4B) and/or the expression of the *copA* efflux pump in all strains (Fig. 4F). The magnitude of this response was to a lesser extent than zinc, which may reflect the limited abundance of copper in the media (0.40 µM). Nevertheless, these data indicate that PBT2 can also elevate cellular copper in *K. pneumoniae* and activate copper efflux responses.

Impacts of PBT2 on cellular manganese differed between strains, with substantial increases only observed for strain Kp661 (Fig. 4C), correlating with decreased expression of the *mntP*-encoded efflux pump (Fig. 4G). PBT2 and zinc have differing impacts on cellular manganese in the literature, with decreases seen in streptococcal species (20, 24), compared with the increases seen here and elsewhere (19). Streptococcal species tend to be more manganese dependent than most Gram-negative species, which often have iron-centric metabolism (32). This may suggest that the different impacts on manganese content are not directly mediated by PBT2, but may be due to cellular management of zinc dyshomeostasis.

PBT2 had a major impact on cellular iron, which was significantly decreased in all strains, irrespective of meropenem exposure (Fig. 4D). This correlates with what has previously been observed in *K. pneumoniae* (19), and to a lesser extent for *A. baumannii* (21) and *S. pyogenes* (20). Detailed understanding of how iron homeostasis systems contribute to this phenotype in *K. pneumoniae* will be complex, as numerous iron transporters and siderophore systems mediate uptake of this essential micronutrient (33), while iron-specific efflux mechanisms are not well understood. However, most strains showed increased expression of the *fhuC* subunit of the FhuABC ferric iron uptake system in the presence of PBT2 (Fig. 4H), consistent with an iron starvation response. For MS6671, we have previously observed *fhuA* expression is increased by PBT2 in the absence of zinc-supplementation (19). This suggests that this system may already be active in the zinc-supplemented media baseline, or there may be strain specific differences in how and when iron limitation responses are activated. Further investigations into the interaction of *K. pneumoniae* zinc and iron homeostatic systems are warranted to elucidate the molecular basis of the observed dysregulation.

**Fig 5:**
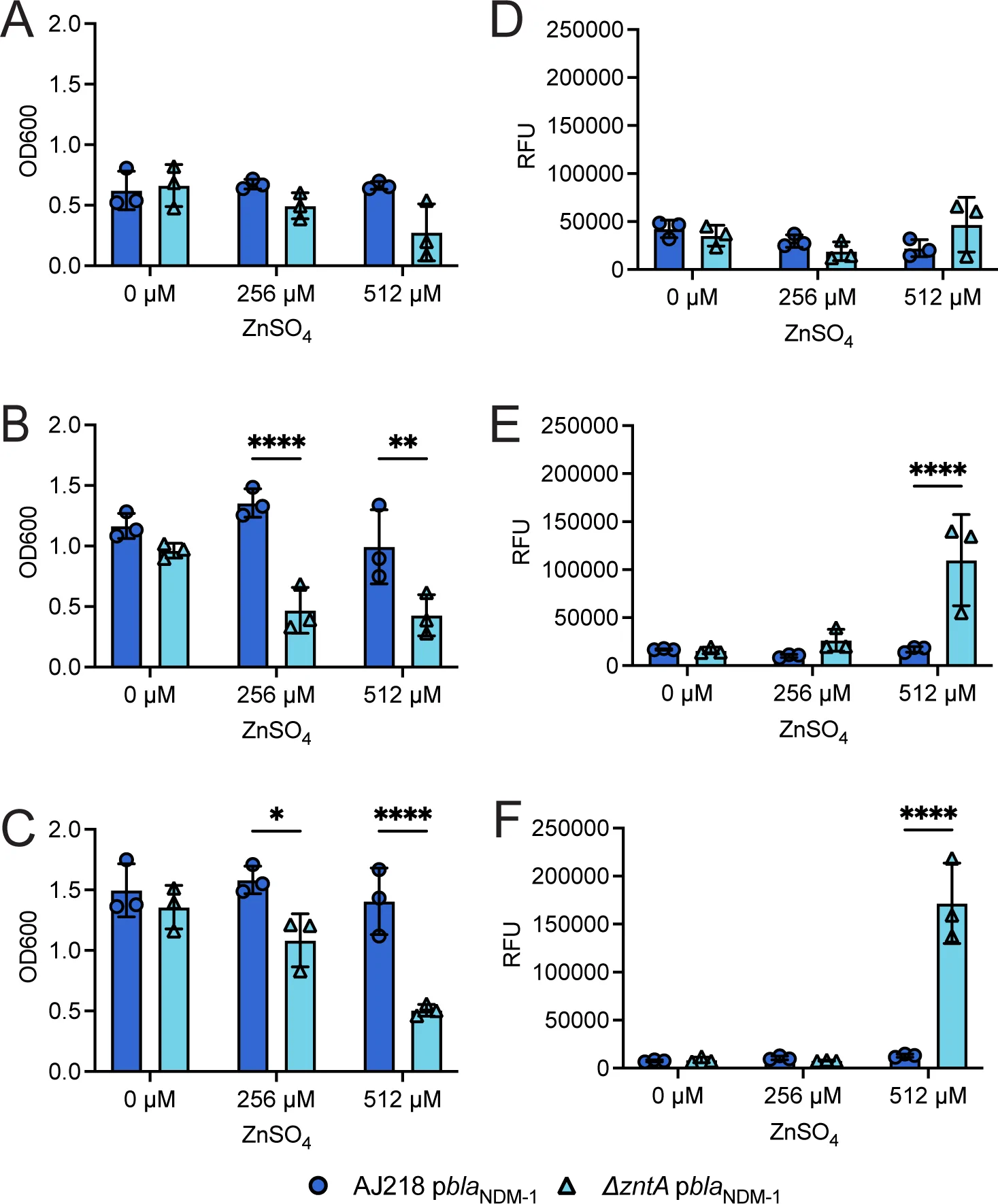
Growth and membrane penetration of AJ218 and ∆*zntA* strains in the presence of ZnSO_4_. *K. pneumoniae* AJ218 and isogenic ∆*zntA* mutant strains grown in CAMHB with or without zinc supplementation (as ZnSO_4_) were analyzed for growth (as OD_600_, **A–C**) and relative fluorescence (with relative fluorescence units [RFU] calculated as fluorescence / OD_600_, **D–F**). Data represent values (±SD) from three independent experiments assessed at 2 (**A and D**), 4 (**B and E**), and 6 (**C and F**) h post-inoculation. Data were obtained from three independent experiments; statistical significance of differences was determined by two-way ANOVA analysis with Tukey post-test. ns, not significant; *, *P* < 0.05; **, *P* < 0.01; ***, *P* < 0.001; ****, *P* < 0.0001.

Notably, prior studies of the *K. pneumoniae* zinc efflux transporter observed that increased manganese and decreased iron also occurred in a Δ*zntA* strain (29), and zinc intoxication has previously been reported to cause decreased β-lactam resistance in other bacterial species, including *A. baumannii*, where susceptibility is linked to carbapenemase downregulation (34), and *S. pneumoniae*, where there is dysfunction of peptidoglycan synthesis (24). This suggests that the inability to manage intracellular zinc stress contributes to the observed phenotypes. To investigate this hypothesis, we assessed whether mutational zinc intoxication in *K. pneumoniae* would cause increased carbapenem sensitivity in the absence of PBT2. For this work, carbapenem resistance was introduced into the AJ218 and isogenic *∆zntA* mutant strains via transformation of the *bla*_NDM-1_ or *bla*_NDM-5_ carbapenemase allele, and meropenem resistance was assessed in increasing concentrations of zinc supplementation. As previously observed, all strains showed maximum meropenem resistance in the presence of zinc supplementation, with higher resistance seen in the AJ218 strains. When zinc concentrations are increased to stress levels in the ∆*zntA* mutant, meropenem resistance in this background is halved, compared with no change in the AJ218 background (Table 4). To assess whether this difference correlated with increased membrane permeability, the AJ218 and ∆*zntA* strains (carrying p*bla*_NDM-1_) were grown in the presence of increasing zinc concentrations. The data from these assays showed that strains grew similarly in the absence of zinc supplementation but that growth of the ∆*zntA* p*bla*_NDM-1_ strain became perturbed by increased levels of zinc (256 and 512 µM ZnSO_4_, Fig. 5A through C). When membrane permeability was assessed, the data showed that there was no significant difference between strains at 0 or 256 µM ZnSO_4_, but ∆*zntA* p*bla*_NDM-1_ strains grown in 512 µM ZnSO_4_ have significantly higher permeability over the course of the assay (Fig. 5D through F). Taken together, these data suggest that the conserved mechanism by which PBT2 impacts *K. pneumoniae* carbapenem sensitivity is through the zinc intoxication of cells, causing dyshomeostasis of cellular metal content and increased permeability to antimicrobial agents.

**TABLE 4:** Minimum inhibitory concentrations of meropenem (µg·mL^−1^) for AJ218 and isogenic ∆*zntA* strain carrying introduced carbapenemase resistance genes in the presence of ZnSO_4_

Plasmid	Strain	ZnSO_4_ concentration^*a*^		
		0 µM	256 µM	512 µM
p*bla*_NDM-1_	AJ218	4	32	32
	∆*zntA*	2	16	8
p*bla*_NDM-5_	AJ218	8	32	32
	∆*zntA*	8	16	8

^*a*^ Minimum inhibitory concentration values as determined by broth microdilution in normal cation-adjusted Mueller–Hinton broth, with added ZnSO_4_ at the concentrations listed. The modal value is presented (from *n* ≥ 3).

### Concluding remarks

Carbapenem antibiotics are a critical line of defense against infections caused by multidrug-resistant Enterobacterales. Resistance to these key drugs is multifactorial, with a mobile genetic pool of carbapenemase enzymes and genomic mutations contributing to rapid rises in resistance despite stewardship measures (1). Current combination therapies to address this crisis frequently pair carbapenems with a β-lactamase inhibitor that directly targets the carbapenemase. While effective, this strategy also has some limitations in use; for example, vaborbactam has potent activity against class A and C β-lactamases (particularly KPC-producing strains) but is less effective against carbapenemases from class B (metallo-β-lactamases) and class D (oxacillinases) and is impacted by porin mutations that reduce its entry into the cell (35). Consequently, recent Clinical and Laboratory Standards Institute (CLSI) guidance (2025 CLSI M100) recommends identification of carbapenemases in Enterobacterales to best inform treatment options (36). This paper describes the potential of the ionophore PBT2 to act as a broad-spectrum carbapenem potentiating agent, increasing the susceptibility of genetically diverse classic and hypervirulent *K. pneumoniae* strains with carbapenemases from all Ambler classes and other resistance determinants. Our data suggest that PBT2 has broad efficacy against diverse *K. pneumoniae* because it does not directly target specific drug resistance mechanisms but rather consistently alters *K. pneumoniae* metal homeostasis and cell membrane stability to permit greater penetration of antimicrobial agents into the cell, as has been seen with tetracycline class antibiotics in *A. baumannii* (21). Given the consistency of PBT2 to chemically induce zinc intoxication in numerous bacterial pathogen species regardless of the presence of normal efflux mechanisms (19, 20, 24, 30, 31), this may provide support for the development of PBT2 as a broad-spectrum potentiator compound that can resensitize bacterial pathogens to multiple drugs. Further screening and mechanistic investigations will provide insight into how widely PBT2 may be deployed as a therapeutic with respect to antimicrobial pairings (and mechanisms of drug resistance, i.e., target protection, compared to drug modification or efflux), bacterial species, and strains.

Understanding how PBT2, zinc, and antibiotics co-localize *in vivo* will provide critical information on where increased penetration and potentiation of antimicrobials can be achieved. The elemental landscape of mammals is complex and dynamic, and in humans, total zinc concentrations range from a mean plasma level of 60–100 µg·dL^−1^ (approximately 10–20 µM) to 16–50 mg·kg^−1^ (242–750 µM) in organ and muscle (37, 38). However, these concentrations vary in response to infection in niche-specific ways. In tissues such as the lung, infections can induce host-mediated zinc intoxication strategies, causing significant localized increases (39, 40). Conversely, withholding of zinc and other metal ions occurs as part of the innate immune response, mediated by host proteins such as albumin and calprotectin that sequester essential metal ion micronutrients. This process, commonly termed nutritional immunity, reduces zinc bioavailability in the bloodstream, impairing the replication of invading pathogens (41, 42). Overall, the *in vivo* efficacy of PBT2 is predicted to be directly influenced by natural host zinc flux, and likely to increase in tissues with higher zinc abundance (offsetting any potential increased activity from MBLs), compared to environments with low zinc bioavailability, such as the serum.

Defining *in vivo* PBT2 concentrations and distribution will require further investigation, and whether host zinc is mobilized by PBT2 from labile carrier molecules, zinc influx, or directly acquired from metal-sequestering proteins remains unknown. However, prior studies have shown that intraperitoneal administration of PBT2 (at a dose of 3.34 mg·kg^−1^ per day, within the dosing range previously established to be safe for humans in clinical trials [43]) potentiated the activity of polymyxin-based antibiotics of disseminated *K. pneumoniae* in the lungs and spleen (19). Similarly, orally delivered PBT2 (15 mg·kg^−1^) can enable antibiotic potentiation and reductions in bacterial burden in lung infections caused by other bacterial pathogens (21, 24). Taken together, these findings highlight that PBT2 can mobilize to niches in the host where there is sufficient zinc for antibiotic potentiation, supporting further development of this potential co-therapeutic approach.

One potential advantage to the development of PBT2 and similar zinc-based therapeutics for combating infectious disease is that there may be less scope for resistance development. The impacts of zinc toxicity on bacterial cells are pleiotropic, as zinc mismetallation (i.e., displacement of cognate co-factors causing protein dysfunction [44]) is a non-specific phenomenon. While this will not impact zinc-binding proteins (such as the metallo-β-lactamases), other metal co-factors, such as iron, are fundamental to the growth and pathogenesis of *K. pneumoniae*. As such, no single mutation is likely to fully confer resistance to zinc toxicity. Moreover, as zinc is also an essential micronutrient for bacteria (42), fitness costs preclude the development of resistance via mutations that would completely exclude these metal ions from the cell. Resistance development may also be limited by the suppressive effects of zinc on hypermutation and gene transfer (45), which we have seen manifested in passage experiments (19) and in gene transfer experiments in this work. Combined with the broad-spectrum mechanism of action of PBT2 and the consistency of metal dyshomeostasis, PBT2 could be an effective and enduring co-therapeutic to combat the dynamic and constantly evolving resistance profiles of *K. pneumoniae* populations.

## MATERIALS AND METHODS

### Bacterial strains, chemicals, and media

Bacterial strains and plasmids used in this study are listed in Tables S4 and S5. *K. pneumoniae* strains were routinely cultured via overnight growth on Luria agar, followed by growth in cation-adjusted Mueller–Hinton broth (CAMHB) with elemental content measured at 0.48 µM ^55^Mn, 9.90 µM ^56^Fe, 0.02 µM ^59^Co, 0.73 µM ^60^Ni, 0.40 µM ^63^Cu, and 5.05 µM ^66^Zn. Culture media were supplemented, where appropriate, with the antibiotic chloramphenicol (Sigma-Aldrich; 20 μg·mL^−1^ for *E. coli* and 80 μg·mL^−1^ for *K. pneumoniae*) or with 150 μM 2-6-diaminopimelic acid. Media were also supplemented with imipenem, meropenem (Glentham Life Sciences), ZnSO_4_ (Sigma-Aldrich), and PBT2 (24) at concentrations specified in the text.

### Genome and bioinformatic analyses

Genomic DNA was extracted from *K. pneumoniae* strains using the QIAGEN DNeasy Blood & Tissue Kit (QIAGEN) using manufacturer protocols for Gram-negative bacteria and cleaned with the Monarch DNA Cleanup Kit (New England Biolabs [NEB]). DNA quality was confirmed by automated electrophoresis using DNA Screentape in a Tapestation system (Agilent), and a minimum of 1 µg of DNA was sent for Oxford Nanopore sequencing (Plasmidsaurus, Oregon). Genomic characterization of *K. pneumoniae* was carried out by upload of genomes to Pathogenwatch, as described in reference 25.

### Minimum inhibitory concentration assays

Antibiotic, PBT2, and zinc MICs were determined using modified CLSI microbroth dilution methods. Briefly, twofold antimicrobial dilution series were set up in CAMHB in 96-well flat-bottom plates (Costar). Wells were supplemented with PBT2 and/or zinc (as ZnSO_4_) at 4× concentration into required wells, then mixed with bacterial cells (standardized by optical density at 600 nm [OD_600_] to 0.02) by pipettor. Plates were incubated with lids in a humidified box at 37°C for 20 h and then assessed for visible growth. For checkerboard assays, plates were set up as described above, using twofold serial dilutions of PBT2, and zinc dispensed into wells at 4× concentrations as specified in the text. Experiments were performed with three biological replicates, and data reported as the modal value.

### Horizontal gene transfer analyses

Horizontal gene transfer assays were conducted as previously described (46). Briefly, overnight cultures of donor and recipient cells were mixed at a 2:1 ratio and spotted onto Luria agar, containing 256 µM ZnSO_4_ and/or PBT2 (at 8 or 16 µM), to initiate conjugation. Plates were dried, incubated at 37°C for 1–24 h, then scraped for resuspension in phosphate-buffered saline (PBS). Transfer rates were assessed by plating of mixes on selective and non-selective agar for the mobilized DNA. For attempts to conjugate native resistance plasmids, donor strains consisted of the clinical strain panel, with a *K. pneumoniae* AJ218 recipient, with incubation times extended to 24 h. For conjugation of the pBRH1 plasmid and the pDS1028 TraDIS transposon mutagenesis plasmid (47), a DAP auxotroph donor *E. coli* (JKE210) was used with recipient AJ218, with an incubation time of 1 h.

### Construction of constitutive carbapenemase expression vectors

Constitutive carbapenemase-expressing vectors were constructed by PCR amplification of pACYC184 and the specified carbapenemase genes and assembled with NEBuilder as per the manufacturer’s protocols (NEB). All carbapenemases were cloned into the pACYC184 *tet* gene sequence to allow constitutive expression from the *tet* promoter. Oligonucleotides for all constructs are listed in Table S5.

### RNA isolation and quantitative real-time PCR analyses

*K. pneumoniae* strains were grown in biological triplicate at 37°C with aeration in CAMHB with 256 µM ZnSO_4_, supplemented with subinhibitory concentrations of PBT2 and/or meropenem, as indicated in the text. At mid-log phase (OD_600_ = 0.6–0.8), cells were harvested by centrifugation (18,000 × *g* for 5 min at room temperature) and then resuspended in RNA Protect Bacteria Reagent (Qiagen). Total RNA was extracted from this sample using the RNeasy Mini Kit in accordance with the manufacturer's instructions. Off-column DNAse I digestion was performed using the RNase-free DNase I set (Qiagen), and removal of genomic DNA contamination was confirmed by PCR. Quantitative real-time PCR was performed on the QuantStudio 7 Real-Time PCR System using the SuperScript III Platinum SYBR Green One-Step qPCR Mix (Thermo Fisher Scientific) using *rpoD* to normalize gene expression, essentially as described previously (29, 48). Oligonucleotides used are listed in Table S5.

### Growth and membrane permeability analyses

*K. pneumoniae* strains were harvested from overnight plate cultures and resuspended to an OD_600_ of 0.1 in CAMHB in the presence and absence of 256 µM ZnSO_4_ or subinhibitory concentrations of PBT2 and/or meropenem, as indicated in the text. Strains were incubated stationary at 37°C in clear-bottom, black-walled 96-well plates (Greiner) for up to 6 h. To assess membrane penetration, the impermeant nucleic acid dye Sytox Green (Thermo Fisher) was added to prepared wells at a concentration of 5 µM and cells incubated 20 min. Plates were read for OD_600_ and fluorescence (excitation at 485 and emission at 520) at times indicated in the text.

### Elemental analyses

Elemental content analyses were performed by inductively coupled plasma–mass spectrometry with an Agilent 8900 ICP-QQQ-MS (Agilent Technologies), using protocols for bacterial elemental analyses described previously (48). Briefly, *K. pneumoniae* strains were subcultured into CAMHB at an OD_600_ of 0.05 and grown with aeration at 220 rpm in biological triplicate at 37°C in the presence and absence of 256 µM ZnSO_4_ or subinhibitory concentrations of PBT2 and/or meropenem, as indicated in the text. At mid-log phase (OD_600_ = 0.6–0.8), cells were harvested by centrifugation at 7,000 × *g* for 10 min at room temperature and then washed (twice in PBS containing 5 mM ethylenediaminetetraacetic acid, followed by two washes in PBS alone). Pellets were desiccated overnight at 95°C, followed by digestion of organic material in 250 μL 65% (vol/vol) HNO_3_ at 95°C for 20 min. Samples were then centrifuged at 18,000 × *g* for 25 min at room temperature, and the supernatant was diluted to a final concentration of 3.25% (vol/vol) HNO_3_ in MilliQ H_2_O. For baseline medium analyses, samples were diluted in a 1:2 with 65% (vol/vol) HNO_3_ and incubated at 95°C for 20 min before centrifugation (18,000 × *g*, 25 min, room temperature) and dilution to 3.25% (vol/vol) HNO_3_ in MilliQ H_2_O for analysis.

### Statistical analyses

Unless otherwise stated, data represent the mean ± standard error of the mean from three independent experiments. Statistical analyses were performed in GraphPad Prism (version 10.4.1), with test details described.

## Data Availability

The data supporting this work are available under BioSample accession numbers SAMN53214200–SAMN53214202.

## SUPPLEMENTAL MATERIAL

Supplemental tables and figure spectrum.02109-26-s0001.pdf Tables S1 to S5 and Fig. S1.

ASM does not own the copyrights to Supplemental Material that may be linked to, or accessed through, an article. The authors have granted ASM a non-exclusive, world-wide license to publish the Supplemental Material files. Please contact the corresponding author directly for reuse.
