# Supplementary material for: PBT2 potentiates carbapenem antibiotics against diverse Klebsiella pneumoniae strains
Source: Microbiol Spectr. 2026 Sep 25;14(11):e02109-26. doi: 10.1128/spectrum.02109-26 (PMC13729099; doi:10.1128/spectrum.02109-26)
Supplement: Supplementary file 1 [file spectrum.02109-26-s0001.pdf]

Contents

Supp. Table 1. Characteristics of *K. pneumoniae* strains used in study.

Supp Table 2. Minimum inhibitory concentrations of PBT2 (µM) against *K. pneumoniae* strains at set concentrations of zinc

Supp Table 3. MICs of AJ218 and B5055 engineered with bla_{KPC}, bla_{IMP}, and bla_{OXA} carbapenemase resistance

Supp Table 4. Strains and plasmids used in this study

Supp Table 5. Oligonucleotides used in this study

Supp Fig. 1. Growth kinetics of cells grown in PBT2 and meropenem

Supplementary Table 1. Characteristics of *K. pneumoniae* strains used in study.

Strain (biosample accession)	MLST[1]	K and O[1] type	Siderophore complement[1]	AMR determinants[1,3]
**AJ218** (SAMEA5128437)	ST2121	K54[2], O1	-	AMG: *aadA*<br>PEN: SHV-27, SHV-44
**B5055** (SAMN18477484)	ST66	K2, O1	*ybt12, iuc2,iro2*	None identified
**MS6671** (SAMEA3257677)	ST147	K64, O2a	-	AMG: *aac(6')-Ib, aac(6')-Ib, aadA2, rmtF, rmtF*<br>CAR: OXA-181 (3 copies) OmpK35-76%, OmpK36TD<br>CEPH: CTX-M-15<br>PEN: SHV-11
**Kp125** (SAMN20033446)	ST258	KL106, O2afg	*ybt13*	AMG: *aac(6')-Ib', aadA2, aph3-Ia*<br>CAR: KPC-2, OmpK35-25%<br>CEPH: SHV-12<br>PEN: TEM-1D, SHV-11
**Kp661** (SAMN53214200)	ST11	K15, O4	*ybt9*	AMG: *aph(3')-VI, armA, strA, strB*<br>CAR: NDM-5, OXA-48, OmpK36GD<br>CEPH: CTX-M-14<br>PEN: SHV-11
**Kp705** (SAMN53214201)	ST11	K2, O2a	*ybt16*	AMG: *aac(3)-IId, aac(6')-Ib-cr, aac(6')-Ib3, rmtC*

				CAR: NDM-1, OXA-181, OmpK35-40%<br>CEPH: CTX-M-15, CMY-6 (3 copies)<br>PEN: OXA-1, SHV-11
**Kp706** (SAMN53214202)	ST8574[4]	KL126, OL101	-	AMG: *aac(3)-IIa, aac(3)-IId, aac(6')-Ib-cr, aac(6')-Ib4, strA, strB*<br>CAR: IMP-4 (homolog), OmpK35-79%<br>CEPH: CTX-M-15<br>PEN: OXA-1, TEM-1D, TEM-1D, SHV-201

1 Data from Pathogenwatch (1) unless otherwise indicated. For K-type, the predicted capsule type given (K), unless listed unknown and K locus (KL) type given instead

2 Capsule type from (2)

3 Resistance determinants for aminoglycosides (AMG), carbapenems (CAR), 3rd generation cephalosporins (CEPH) and penicillins (PEN)

4 Novel MLST type, assigned by Pathogenwatch (1)

Supplementary Table 2. Minimum inhibitory concentrations of PBT2 (µM) against *K. pneumoniae* strains at set concentrations of zinc

Strain	ZnSO$_4$ (µM) [1]							
	**512**	**256**	**128**	**64**	**32**	**16**	**8**	**0**
**AJ218**	8-16	16	32	64	64	128	256	256
**B5055**	8	8-16	16	32	32	64	64-128	256
**MS6671**	128	128	128	256	256	>256	>256	>256
**Kp125**	64	64	128	128	256	256	>256	>256
**Kp661**	32	32	32-64	64	128-256	256	>256	>256
**Kp705**	64	64	128	256	>256	>256	>256	>256
**Kp706**	16	16-32	32	32	64	128	256	>256

[1] Minimum inhibitory concentration (MIC) values were determined by broth microdilution in normal cation-adjusted Mueller Hinton broth, according to (3), with added ZnSO$_4$ at concentrations listed. Modal value is presented (from $n \geq 3$).

Supplementary Table 3. Minimum inhibitory concentrations of meropenem (µg/mL) for lab strains carrying trans-expressed carbapenemase resistance genes in the presence of PBT2 and $ZnSO_4$

Plasmid	Strain	Assay conditions[1]			
		Low zinc (5.05 µM $ZnSO_4$)		Zinc-supplemented (256 µM $ZnSO_4$)	
		-PBT2	+ PBT2	-PBT2	+ PBT2
**p*bla*$_{\mathbf{IMP}}$**	**AJ218**	<0.5	<0.5	<0.5	<0.5
	**B5055**	<0.5	<0.5	<0.5	<0.5
**p*bla*$_{\mathbf{KPC}}$**	**AJ218**	4	2-4	2-4	2
	**B5055**	4	1-2	2-4	1
**p*bla*$_{\mathbf{OXA\text{-}48}}$**	**AJ218**	<0.5	<0.5	<0.5	<0.5
	**B5055**	<0.5	<0.5	<0.5	<0.5
**p*bla*$_{\mathbf{OXA\text{-}181}}$**	**AJ218**	<0.5	<0.5	<0.5	<0.5
	**B5055**	<0.5	<0.5	<0.5	<0.5

[1] Minimum inhibitory concentration (MIC) values as determined by broth microdilution in normal cation-adjusted Mueller Hinton broth (no $ZnSO_4$, no PBT2 column), and with treatments as indicated ($ZnSO_4$ at 256 µM and PBT2 at concentrations listed). Modal value is presented (from n ≥ 3).

[2] PBT2 used at subinhibitory levels: 4 µM for strain B5055, and 8 µM for AJ218

Supplementary Table 4. Strains and plasmids used in this study

Strain	Genotype and/or description	Reference
**AJ218**	Wild-type *Klebsiella pneumoniae* clinical isolate, serotype K54, Ap^R	(4)
**B5055**	Wild-type *K. pneumoniae* clinical isolate*;* mouse lethal*,* serotype K2:O1 Ap^R	(5)
**MS6671**	Multi-drug resistant clinical *K. pneumoniae* isolate, serotype K6:O2a carbapenem resistant (*bla*$_{\text{OXA-181}}$) [1]	(6)
**AUSMDU00012530 (Kp125)**	Clinical *K. pneumoniae* strain, serotype KL106:O2afg, carbapenem resistant (*bla*$_{\text{KPC-2}}$)[1]	BioProject PRJNA52944
**AUSMDU00066103 (Kp661)**	Clinical *K. pneumoniae* strain, serotype K15:O4, carbapenem resistant (*bla*$_{\text{NDM-5}}$, *bla*$_{\text{OXA-48}}$) [1]	This study (BioProject PRJNA857533)
**AUSMDU00070561 (Kp705)**	Clinical *K. pneumoniae* strain, serotype K2:O2a, carbapenem resistant (*bla*$_{\text{NDM-1}}$, *bla*$_{\text{OXA-181}}$) [1]	This study (BioProject PRJNA857533)
**AUSMDU00070655 (Kp706)**	Clinical *K. pneumoniae* strain, serotype KL126:OL101, carbapenem resistant (*bla*$_{\text{IMP-4}}$) [1]	This study (BioProject PRJNA857533)
**AJ218 Δ*zntA***	AJ218 with deletion of *zntA*	(7)
**AJ218ΔOmpK35**	AJ218 with deletion of *ompK35*	(8)
**AJ218ΔOmpK36**	AJ218 with deletion of *ompK36*	(8)
**AJ218ΔOmpK35 ΔOmpK36**	AJ218 with deletion of *ompK35* and *ompK36*	(8)

**NEB5-α**	High efficiency competent *E. coli*, *fhuA2* *(argF-lacZ)*U169 *phoA* *glnV44* 80 *(lacZ)*M15 *gyrA*96 *recA1* *relA1* *endA1* *thi-1* *hsdR17*	New England Biolabs
**JKE210**	*E. coli* DAP auxotroph	(9)
**Plasmid**	**Feature or description**	**Reference**
**pACYC184**	Low-copy-number cloning vector, p15A ori; Tetracycline^{R} Chl^{R}	New England Biolabs
**p*bla*$_{\mathbf{IMP}}$**	pACYC184 vector constitutively expressing *bla*$_{IMP-4}$ allele from pTet promoter	This study
**p*bla*$_{\mathbf{KPC}}$**	pACYC184 vector constitutively expressing *bla*$_{KPC-2}$ allele from pTet promoter	This study
**p*bla*$_{\mathbf{NDM-1}}$**	pACYC184 vector constitutively expressing *bla*$_{NDM-1}$ allele from pTet promoter	This study
**p*bla*$_{\mathbf{NDM-5}}$**	pACYC184 vector constitutively expressing *bla*$_{NDM-5}$ allele from pTet promoter	This study
**p*bla*$_{\mathbf{OXA-48}}$**	pACYC184 vector constitutively expressing *bla*$_{OXA-48}$ allele from pTet promoter	This study
**p*bla*$_{\mathbf{OXA-181}}$**	pACYC184 vector constitutively expressing *bla*$_{OXA-181}$ allele from pTet promoter	This study
**pBHR1**	Broad host range mobilizable vector, Chl^{R}	MoBiTech
**pDS1028**	plasmid-transposon hybrid mutagenesis system, Chl^{R}	(10)

[1]Further resistance determinants listed in Supplementary Table 1

Supplementary Table 5. Oligos used in this study

Name	Sequence (5' → 3')	Purpose
pACYC184_F2	CGGTGCCTGACTGCGTTAG	Complementation: pACYC184 amplification for constitutive gene expression
pACYC184_R	GCGAGAAGCAGGCCATTATCG	
pACYC_seq_F	GCTAACGCAGTCAGGCACC	Sequencing primers for checking pACYC184 inserts
pACYC_seq_R	CAGCAAGACGTAGCCCAGC	
IMP_comp_F	CTAACGCAGTCAGGCACCG AGTATGAGCAAGTTATCTGTATTC	Complementation: *bla*$_{IMP}$ (from Kp706) amplification for expression in pACYC184
IMP_comp_R	CGATAATGGCCTGCTTCTCGC TTAGTTGCTTAGTTTTGATGGTT	
KPC_comp_F	CTAACGCAGTCAGGCACCG TTGATGTCACTGTATCGCCGTC	Complementation: *bla*$_{KPC}$ (from Kp125) amplification for expression in pACYC184
KPC_comp_R	CGATAATGGCCTGCTTCTCGC TTACTGCCCGTTGACGCC	
NDM_comp_F	CTAACGCAGTCAGGCACCG TTGATGGAATTGCCCAATATTATGCAC	Complementation: *bla*$_{NDM}$ (from Kp661 and Kp705) amplification for expression in pACYC184
NDM_comp_R	CGATAATGGCCTGCTTCTCGC TCAGCGCAGCTTGTCGGC	
OXA_comp_F	CTAACGCAGTCAGGCACCG GTTATGCGTGTATTAGCCTTATCG	Complementation: *bla*$_{OXA}$ (from Kp661 and Kp705) amplification for expression in pACYC184
OXA_comp_R	CGATAATGGCCTGCTTCTCGC CTAGGGAATAATTTTCTCCTGTTTGAG	
rpoD562F	GAAGAGATGGATGACGACGAAGACG	Real time qPCR: housekeeping control (4)
rpoD677R	GTACGCAGCTCGGCGAATTTCTCAC	

OXA_rt_F	CGGTAGCAAAGGAATGGCAAG	Real time qPCR: *bla*$_{OXA}$
OXA_rt_R	CCTTGCTGCTTATTCTCATTCCAG	
NDM_rt_F	GAAGCTGAGCACCGCATTAG	Real time qPCR: *bla*$_{NDM}$
NDM_rt_R	CCAGATCGCCAAACCGTTG	
IMP_rt_F	GACACTCCATTTACGGCTAAAGATAC	Real time qPCR: *bla*$_{IMP}$
IMP_rt_R	CACTCTATTCCGCCCGTGC	
KPC_rt_F	TCTGCTGTCTTGTCTCTCATGG	Real time qPCR: *bla*$_{KPC}$
KPC_rt_R	GGTATCCATCGCGTACACACC	
zntA_rt_F2	CAATTCTCGTCTTTCACCATGCG	Real time qPCR: *zntA*
zntA_rt_R2	GCCATCAACTTGCCAGCTATAG	
copA_rt_F	CACTATCGACCTGACGCTGG	Real time qPCR: *copA*
copA_rt_R	GTGACTTCCGCTTGCTCAAC	
mntP_rt_F	GCTACCATTCTTCTCGCTTTCG	Real time qPCR: *mntP*
mntp_rt_R	CGTACTGCCTCTGAAAATTTGGG	
fhuC_rt_F	CACATTGCTCCATCCGCTC	Real time qPCR: *fhuC*
fhuC_rt_R	GATGACGGCCAAGCATTTTCAG	
acrA_rt_F	GTGACGCTCAAATCCGCAC	Real time qPCR: *acrA*
acrA_rt_R	CACTGACCTGAGGACGGAC	

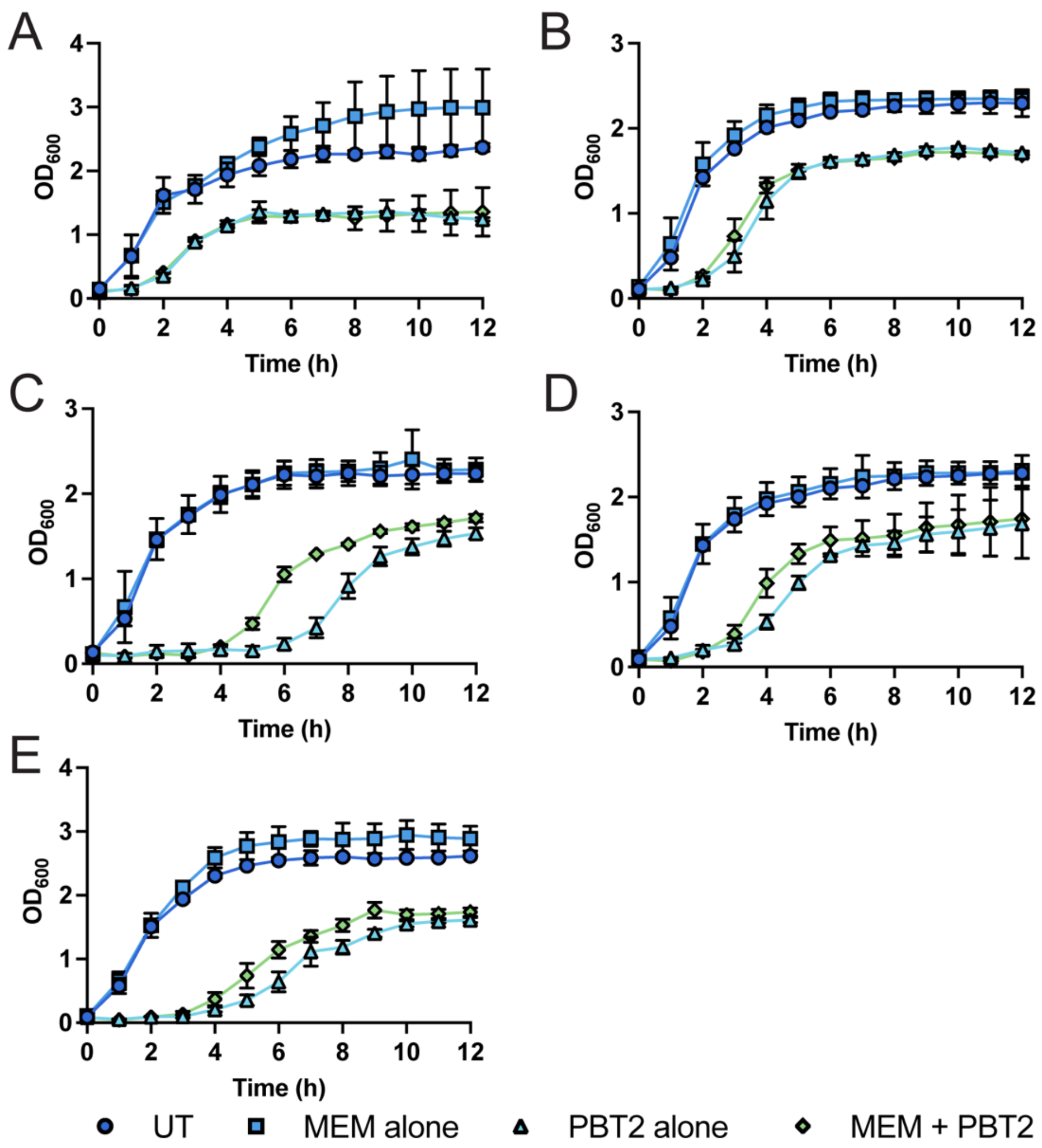

Supplementary Figure 1. Growth kinetics of cells grown in PBT2 and meropenem

K. pneumoniae strains grown zinc-supplemented CAMHB with or without subinhibitory PBT2 (0.5 × MIC in 256 μM $ZnSO_4$) and meropenem (0.25 × MIC, relative to PBT2+Zn), analysed for growth kinetics for MS6671 (**A**), Kp125 (**B**), Kp661, (**C**), Kp705 (**D**), and Kp706 (**E**), respectively. Data represent values (± SD) from three independent experiments.

References

1. Argimón S, David S, Underwood A, Abrudan M, Wheeler NE, Kekre M, Abudahab K, Yeats CA, Goater R, Taylor B, Harste H, Muddyman D, Feil EJ, Brisse S, Holt K, Donado-Godoy P, Ravikumar KL, Okeke IN, Carlos C, Aanensen DM, Resistance NGHRUoGSoA. 2021. Rapid genomic characterization and global surveillance of *Klebsiella* using Pathogenwatch. Clin Infect Dis 73:S325-S335.
2. Wilksch JJ, Yang J, Clements A, Gabbe JL, Short KR, Cao H, Cavaliere R, James CE, Whitchurch CB, Schembri MA, Chuah MLC, Liang Z-X, Wijburg OL, Jenney AW, Lithgow T, Strugnell RA. 2011. MrkH, a Novel c-di-GMP-Dependent Transcriptional Activator, Controls *Klebsiella pneumoniae* Biofilm Formation by Regulating Type 3 Fimbriae Expression. PLoS Pathog 7:e1002204.
3. Bellio P, Fagnani L, Nazzicone L, Celenza G. 2021. New and simplified method for drug combination studies by checkerboard assay. Methods X 8.
4. Wilksch JJ, Yang J, Clements A, Gabbe JL, Short KR, Cao H, Cavaliere R, James CE, Whitchurch CB, Schembri MA, Chuah MLC, Liang ZX, Wijburg OL, Jenney AW, Lithgow T, Strugnell RA. 2011. MrkH, a novel c-di-GMP-dependent transcriptional activator, controls *Klebsiella pneumoniae* biofilm formation by regulating type 3 fimbriae expression. PLoS Pathog 7.
5. Clements A, Tull D, Jenney AW, Farn JL, Kim S-H, Bishop RE, McPhee JB, Hancock REW, Hartland EL, Pearse MJ, Wijburg OLC, Jackson DC, McConville MJ, Strugnell RA. 2007. Secondary Acylation of Klebsiella pneumoniae Lipopolysaccharide Contributes to Sensitivity to Antibacterial Peptides. J Biol Chem 282:15569-15577.
6. Zowawi HM, Forde BM, Alfaresi M, Alzarouni A, Farahat Y, Chong T-M, Yin W-F, Chan K-G, Li J, Schembri MA, Beatson SA, Paterson DL. 2015. Stepwise evolution of pandrug-resistance in Klebsiella pneumoniae. Sci Rep 5:15082.
7. Maunders EA, Ganio K, Hayes AJ, Neville SL, Davies MR, Strugnell RA, McDevitt CA, Tan A. 2022. The role of ZntA in *Klebsiella pneumoniae* zinc homeostasis. Microbiol Spectr 10:e01773-21.
8. Rocker A, Lacey JA, Belousoff MJ, Wilksch JJ, Strugnell RA, Davies MR, Lithgow T. 2020. Global trends in proteome remodeling of the outer membrane modulate antimicrobial permeability in *Klebsiella pneumoniae*. mBio 11:e00603-20.
9. Harms A, Liesch M, Körner J, Québatte M, Engel P, Dehio C. 2017. A bacterial toxin-antitoxin module is the origin of inter-bacterial and inter-kingdom effectors of Bartonella. PLoS Genet 13:e1007077.
10. Monson R, Smith DS, Matilla MA, Roberts K, Richardson E, Drew A, Williamson N, Ramsay J, Welch M, Salmond GPC. 2015. A plasmid-transposon hybrid mutagenesis system effective in a broad range of Enterobacteria. Front Microbiol Volume 6 - 2015.
